# Optimized preparation of activated carbon with high porosities based on puck shells (*Afrostyrax lepidophyllus*) by response surface methodology and physico-chemical characterization

**DOI:** 10.1098/rsos.230911

**Published:** 2023-12-06

**Authors:** Gédéon Nzetchuen Kouahou, Cyrille Ghislain Fotsop, Liouna Adoum Amola, Cyrille Donlifack Atemkeng, Arnaud Kamdem Tamo, Georges Teikam Kenda, Rufis Fregue TIEGAM TAGNE, Theophile Kamgaing

**Affiliations:** ^1^ Research Unit of Noxious Chemistry and Environmental Engineering, Department of Chemistry, University of Dschang, Dschang, Cameroon; ^2^ Institute of Chemistry, Faculty of Process and Systems Engineering, Universität Platz 2, 39106 Magdeburg, Germany; ^3^ Laboratory for Bioinspired Materials BMBT, Institute of Microsystems Engineering IMTEK, University of Freiburg, 79104 Freiburg, Germany; ^4^ Freiburg Center for Interactive Materials and Bioinspired Technologies FIT, University of Freiburg, 79104 Freiburg, Germany; ^5^ Freiburg Materials Research Center FMF, University of Freiburg, 79104 Freiburg, Germany; ^6^ Department of Paper Sciences and Bioenergy, University Institute of Wood Technology, University of Yaounde I, Mbalmayo, Cameroon

**Keywords:** activated carbon, Box–Behnken design, optimized, puck shells

## Abstract

From recent research, lignocellulosic materials assert themselves as good precursors for the manufacture of highly carbonaceous and porous materials. Hence, the perspective of this work is the preparation of the low-cost activated carbons (ACs) based on puck shells (*Afrostyrax lepidophyllus*) with a large surface area. To achieve this, chemical activation using phosphoric acid and sodium hydroxide as an activating agent was carried out. The response surface methodology through Box–Behnken design was used to optimize the preparation conditions. Box–Behnken design was used to optimize the preparation conditions. The factors whose influences have been studied are the concentration of activating agent (0.5–1.5 mol l^−1^), the carbonization temperature (300–500°C) and the residence time (30–100 min). The AC obtained by phosphoric acid was named CRP and impregnated with sodium hydroxide CRB. The ideal conditions for the preparation of the ACs obtained from the maximum iodine number (647.29 for CRP and 575.15 mg g^−1^ for CRB) were: 1.5 mol l^−1^ for the concentration of activating agent at a carbonization temperature of 500°C during 62 min for CRP and 0.85 mol l^−1^ for the concentration of activating agent at a carbonization temperature of 500°C during 59 min for CRB. These two materials prepared were characterized by several techniques pH at the point of zero charge (pHpzc), Boehm titration, Fourier transform infrared spectroscopy, XRD analysis, BET method, Raman spectroscopy and scanning electron microscopy, which confirmed the acidic nature of CRP and the basic nature of CRB carbons. The specific surface of the micropores was 509.05 and 27.53 m^2^ g^−1^, respectively. It provides a new valorization of agricultural waste for the preparation of effective-cost microporous ACs as an adsorbent that can be applied in water treatment industries.

## 1. Introduction

Increase in industrialization and agricultural activities presents a lot of advantages but also some disadvantages in the sense that it generates pollutants of a different nature. These pollutants often end up in the environment and their management remains a problem for scientists. Faced with this problem, several pollution control techniques have been implemented. Due to its accessible cost, its easy implementation, the availability of the raw material and its effectiveness in eliminating low concentration pollutants, researchers are now looking into adsorption using activated carbons (ACs) [1]. ACs are widely used in environmental remediation techniques. This is because of the large specific areas they possess and their highly developed porosity which allow the adsorption of pollutants, whether organic or inorganic in nature [2]. They can be prepared from any material with a high carbon percentage and low percentage of inorganic matter [3,4]. Several studies sufficiently demonstrate the performance of ACs prepared from lignocellulosic materials such as rice husk [5–7], oil palm ash [8], karanj (*Pongamiapinnata*) fruit hulls [9], cola nut (*Cola acuminata*) shells [10], bitter kola (*Garcinia kola*) nut shells [11] and mixture of *Ayous* sawdust and C*ucurbitaceae* peelings [12], shea residue [13] and safou seeds [14]. The use of these precursors for the preparation of AC has a double advantage, firstly in the production of effective materials for water pollution control, and secondly in solving the problem of their storage in the environment and thus enhancing these agro-food residues. It is with this in mind that puck shells were used as a precursor for the preparation of ACs because of their abundance in Cameroon. ACs can be manufactured by chemical or physical activation. In this work, chemical activation has been used because of its lower energy consumption and well-developed porosity of AC obtained. Activating agents which are generally used are phosphoric acid (H_3_PO_4_) [15]; sulfuric acid [16]; zinc chloride [17]; potassium hydroxide [18]; sodium hydroxide (NaOH) [19] and iron (II) sulfate heptahydrate [20]. Herein, H_3_PO_4_ and NaOH were used for the preparation of the ACs. The preparation of an AC with well-developed pores and large specific surface requires optimization of the factors that influence its characteristics. The optimization of ACs has long been done in a classical manner, therefore, requiring more experience and giving less information such as the effect of interactions between the different variables on the desired yield. In fact, varying the parameters in turn leads to a loss of time and chemical reagents [21]. Response surface methodology (RSM) is an alternative that saves time and better predicts results. It is a great alternative for optimizing the preparation of ACs because it allows maximum information to be extracted with little experimentation. RSM is also a statistical tool that provides information on the synergistic and antagonistic effects of different variables during a process [21]. This is why the Box–Behnken design of the RSM was used to obtain maximum information with minimal experiments. This work, therefore, provides an approach to the green synthesis at moderate temperature and concentration of a new cost-effective microporous material derived from puck shell. The objective of this work was to optimize the preparation of ACs based on *Afrostyrax lepidophyllus* shells (CRN) as a precursor which will contribute to the depollution of the environment and also to the valorization of these precursors considered as waste.

## 2. Material and methods

### 2.1. Materials and chemical reagents

Puck shells used as raw material for the production of ACs were collected from Dschang, Menoua division in the West region of Cameroon. NaOH (purity 97%) was purchased from Fischer Scientific; H_3_PO_4_ (purity 68%) was purchased from Fischer Scientific, sodium chloride (NaCl, purity 99.5%) was purchased from Fischer Scientific, hydrochloric acid (HCl, purity 35%) was purchased from Phillip Harris, potassium iodide (KI, purity 99,5%) was purchased from Kem Light Laboratories PVT, iodine (I_2_, 99.9%) was purchased from Labtech Chemicals.

### 2.2. Preparation of activated carbon

Puck shells were collected and washed with tap water to get rid of water-soluble impurities and then dried in an oven for 24 h at 105°C to remove mould and volatile impurities. The puck shells were finally ground and sieved to obtain particles of size 1.5 mm that were preserved for use in this study.

**Figure 1.**
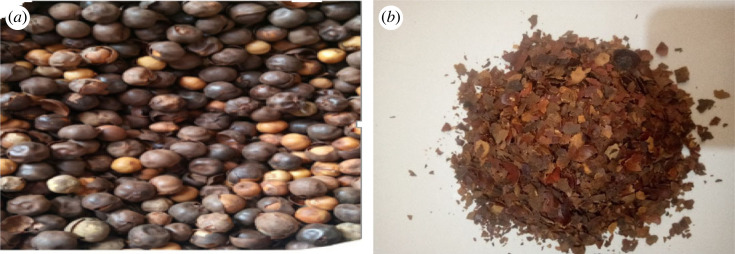
Photograph of *Afrostyrax lepidophyllus* fruit: (*a*) fresh fruits and (*b*) crushed shells.

Chemical activation was used in this study to prepare ACs. For this purpose, H_3_PO_4_ and NaOH were used as activating agents. The preparation of ACs was carried out as follows: 40 g of the crushed and dried CRN were mixed with 40 ml of activating agents of concentrations ranging from 0.5 to 1.5 mol l^−1^. After 24 h of agitation, filtration was carried out and the impregnated CRN was first dried in a Heraeus brand electric oven. The values of the different carbonization factors are between 300 and 500°C for the carbonization temperatures and between 30 and 100 min for residence time. The product obtained was washed with distilled water to remove ashes that had formed during the carbonization process. Finally the material obtained was dried in an oven at 105°C for 24 h and then well crushed to have particles of about 100 µm in diameter. Figure 1 presents the photograph of the fruits of *A. lepidophyllus* fresh fruits and crushed shells.

### 2.3. Methodology of experimental design

An experimental design is used to have the respective influence of the various parameters and their interactions on the design and manufacture of a product [22]. In this study, the Box–Behnken design was chosen and the three factors investigated were carbonization temperature (300–500°C), concentration of the activating agent (0.5–2 mol l^−1^) and carbonization time (30–100 min), and the response analysed was the iodine number of the obtained ACs. The experimental design matrix generated by Statgraphics 5 plus software is presented in electronic supplementary material, table S1. In this work, the activating reagents used were H_3_PO_4_ and NaOH. The RSM was used to develop a model which correlates the amount of iodine adsorbed to the three factors using a mathematical equation given by equation (2.1) [23].

2.1 $$Y=a_{0}+\overset{n}{\underset{i=1}{\sum }}a_{i}x_{j}+\overset{n}{\underset{i,j=1\;\;i\neq j}{\sum }}a_{ij}x_{j}.x_{i}+\overset{n}{\underset{i,j,k=1\;\;i\neq j\neq k}{\sum }}a_{ijk}x_{j}.x_{i}.x_{k}+\varepsilon ,$$

where *Y* is the predicted response (the iodine number), *a*_0_ is the constant coefficient, *a_i_* is the linear coefficients, *a_ii_* is the quadratic coefficients, *a_ij_* is the interaction coefficients, *xi*, *xj* are the coded values of the variables and *ε* the uncertainty between measured and predicted values. The results of the experimental design were studied and analysed by statistical software Statgraphics plus 5.0 to optimize the variables in the carbonization process. Electronic supplementary material, table S2 presents independent experimental variable.

### 2.4. Characterization of activated carbon

#### 2.4.1. Mass yield of the carbonization, iodine number, pH, pH at point of zero charge, moisture content, bulk density of AC and quantification of surface functions by Boehm's method

This is an important quantitative characteristic of ACs which reflects the mass loss of biomass during the carbonization process. The yield of ACs is determined as the weight of the coal relative to the raw material according to equation (2.2)

2.2 $$\mathrm{Yield}=\frac{\mathrm{weight}\;\mathrm{of}\;\mathrm{activated}\;\mathrm{carbon}\;(g)}{\mathrm{weight}\;\;\mathrm{of}\;Afrostyrax\;lepidophyllus\;(g)}\;\times \;100.$$

The American Society of Test and Materials (ASTM) method D4607-94 was used to determine the iodine number of the AC samples produced [24,25]. It measures the micropores contained in the AC. This was done by mixing 30 ml of iodine solution (0.02 N) with 0.1 g of AC. The mixture was stirred for 3 h and then filtered. After filtration, 10 ml of the filtrate were titrated with a sodium thiosulfate solution of concentration of 0.005 N using starch as an indicator. The following equations were used to determine the final iodine concentration.

2.3 $$2{\mathrm{NaS}}_{2}O_{3}(\mathrm{aq})+I_{2}\to {\mathrm{NaS}}_{4}O_{6}(\mathrm{aq})+2\mathrm{NaI}.$$

The amount of iodine adsorbed is determined by the equation (2.4):

2.4 $$Q_{\mathrm{ads}}=\frac{(C_{0}-(C_{T*V_{T}}/2V_{I_{2}}))M_{I_{2\;}}.V_{\mathrm{ads}}}{m},$$

*Q*_ads_ (mg g^−1^) is the mass of iodine adsorbed per gram of adsorbent; *m* (g) is the mass of activated carbon; *C*_0_ (mol l^−1^) is the initial concentration of I_2_ solution; *C_T_* (mol l^−1^) is the concentration of sodium thiosulfate; *V_T_* (ml) is the volume of sodium thiosulfate; *V*_ads_ (ml) is the volume of iodine solution that was mixed with the activated carbon; $V_{I_{2}}$ (ml) is the volume of iodine solution titrated and $M_{I_{2\;}}$ (g mol^−1^) the molar mass of I_2_.

The pH of the ACs was determined by bringing 30 ml of distilled water into contact with a mass of 100 mg of AC in a bottle. The whole is carried under stirring for 24 h. Once decanted, the solution was filtered and the pH of the filtrate was measured with a METTLER TOLEDO model pH meter.

The pH at the point of zero charge (pHpzc) is defined as the pH at which the AC is electrically neutral in solution. That is, the sum of the charges on the surface is zero. The method for determining the pHpzc consists of preparing 0.1 M NaCl solutions at different pH values of 2, 4, 6, 8, 10. pH values were adjusted with a METTLER TOLEDO model pH meter using solutions of NaOH and HCl. Thus, 0.1 g of AC was brought into contact with 30 ml of each solution. The mixture was stirred for 24 h. The suspension was filtered and the pH of the filtrate measured with the pH meter. The curve is obtained by plotting pH*_f_* = *f*(pH*_i_*). The point of intersection between this curve and the first bisector gives the pHpzc of the AC considered.

The moisture content determination followed the following protocol: a dry empty test tube was first weighed and the mass obtained was collected and noted *w*_0_. A mass of each AC was introduced into this test tube (the total mass was noted *w*_1_) and heated for 2 h at 105°C in an oven. After cooling in a dessicator, the mixture was weighed and noted *w*_2_. The humidity level is given by the following equation (2.5)

2.5 $$\text{\%}T=\frac{w_{2}-w_{0}}{w_{1}-w}\times 100.$$

The quantification of the surface functions was carried out according to the Boehm method which corresponds to the acid–base titration of the surface functional groups [26]. 0.1 g of AC was brought into contact with 30 ml of basic solutions (NaOH, Na_2_CO_3_ and NaHCO_3_ each of concentration 0.1 mol l^−1^) and HCl of concentration 0.1 mol l^−1^, respectively, for determination of acid and basic functions on the surface of ACs. The whole was stirred for 24 h at a constant speed of 200 rpm and then filtered. The excess base or acid was titrated using a solution of HCl or NaOH of concentration 0.1 mol l^−1^. Equivalents are calculated using the equation (2.6) below:

2.6 $$n_{\mathrm{eq}}R=N_{i}V_{i}\text{–}N_{f}V_{i},$$

where *n*_eq_*R* represents the reacted gram equivalent number; *N_i_V_i_* is the number of gram equivalent reacted before the reaction and *N_f_V_i_* is the number of gram equivalent reacted after the reaction. Apparent density of the ACs was achieved as follows; the vacuum flask was weighed (*m*_1_). The ACs were then introduced into the vials up to a volume of 5 ml. The filled bottle was weighed (*m*_2_) and the different masses are recorded. The formula (2.7) used to determine bulk density is

2.7 $$d_{\mathrm{app}}=\frac{m_{2}-m_{1}}{V},$$

where *d*_app_ is the bulk density, *m*_1_ is the mass of the vacuum flask, *m*_2_ is the mass of the flask filled with AC and *V* is the volume of the flask used.

#### 2.4.2. Physico-chemical methods for characterizing of AC

The prepared ACs (CRP and CRB) were characterized by a Nicolet ISS0 IR-TF spectrophotometer from Thermo Fischer Scientific. The Fourier-transform infrared spectra of the ACs were recorded from samples in the wavenumber range of 4000–100 cm^−1^.

The identification of the elements and their concentrations in the materials were determined by EDX analysis using a XL30 ESEM from FEI, Hillsboro, USA with a Genesis energy dispersive X-ray detector from EDAX, Mahwah, USA.

The acquisition of the diffraction patterns was carried out on an Empyrean X-ray powder diffractometer from panalytical, Almelo, Netherlands with a copper anode as source of X-ray (characteristic wavelength *λ* = 1.540598 A^°^ and a linear detector coupled to a monochromator).

Raman spectra of different AC were recorded with a Raman spectrometer Almega X/Thermo using laser excitation line at 532 nm.

Surface morphology of the raw material (*A. lepidophyllus*) and the AC prepared under the optimal conditions were analysed by this technique using an ESEM XL30 microscope.

The Brunauer–Emmet–Teller (BET) model was applied to fit nitrogen adsorption isotherm and evaluate the surface area of the sorbent. The measurements are carried out on a volumetric device of the Thermo Electron Corporation brand, and of Sorptomatic Advanced Data Processing which uses nitrogen as an adsorbate.

## 3. Results and discussion

### 3.1. Experimental design

The influence of three factors was carried out in order to optimize this AC synthesis. These factors were concentration (A), temperature (B) and time (C). The influence of these parameters was carried out on the iodine number which served as a response. This optimization has been achieved by experimental research methodology through the Box–Behnken model.

The iodine number permits us to have an appreciation of the microporosity of the material. The results of the iodine number of CRP and CRB obtained are presented in tables 1 and 2, respectively. The mathematical model materializing the variation in iodine number of CRP and CRB carbons is reported in equations (3.1) and (3.2), respectively.

3.1 $$\begin{array}{rl} Y_{1}=\; & 1340.33\text{–}40.6912A\text{–}8.35191B+7.53573C+61.0767A^{2}+0.27795\mathrm{AB}\\ & \text{–}2.04957\mathrm{AC}+0.0119687B^{2}+0.00302143\mathrm{BC}\text{–}0.0489048C^{2} \end{array}$$

and

3.2 $$\begin{array}{rl} Y_{2}= & 347.336+494.272A-1.21972B+2.10491C-158.234A^{2}\\ & \text{–}0.143375\mathrm{AB}\text{–}2.58543\mathrm{AC}+0.0018344B^{2}+0.00705357\mathrm{BC}\\ & \;-0.0277672C^{2} \end{array}$$

**Table 1.** Experimental design matrix for CRP preparation using Box–Behnken design. Exp val, experimental value; pred val, predicted value.

order of experiments	parameters			iodine number *Y*_1_ (mg g^−1^)	
	A (mol l^−1^)	B (°C)	C (min)	exp val	pred val
1	1	400	65	276.23	274.65
2	1	400	65	271.48	274.65
3	1	400	65	276.23	274.65
4	1.5	500	65	647.00	622.95
5	0.5	400	100	276.00	247.96
6	0.5	300	65	200.00	224.05
7	0.5	400	30	150.15	152.63
8	1	300	30	190.00	163.47
9	1	500	30	485.77	481.79
10	0.5	500	65	534.23	535.73
11	1.5	300	65	257.18	255.68
12	1	500	100	500.00	526.54
13	1.5	400	30	255.75	283.79
14	1.5	400	100	238.13	235.65
15	1	300	100	161.93	165.91

**Table 2.** Experimental design matrix for CRB preparation using Box–Behnken design. Exp val, experimental value; pred val, predicted value. *R*^2^ = 97.9107% *R*^2^ adjusted = 94.15% * *=* *significant* d.f. *=* degree of freedom.

number of experiments	parameters			iodine number *Y*_2_ (mg g^−1^)	
	A (mol l^−1^)	B (°C)	C (min)	exp val	pred val
1	1	400	65	466.00	466.48
2	1	400	65	476.25	466.48
3	1	400	65	457.20	466.48
4	1.5	500	65	457.20	467.88
5	0.5	400	100	409.58	420.26
6	0.5	300	65	419.00	408.32
7	0.5	400	30	419.10	413.16
8	1	300	30	447.00	463.62
9	1	500	30	532.25	532.25
10	0.5	500	65	523.88	529.82
11	1.5	300	65	381.00	375.06
12	1	500	100	504.00	487.38
13	1.5	400	30	466.73	456.05
14	1.5	400	100	276.23	282.17
15	1	300	100	320.00	341.70

Let us mention that when the positive sign preludes a factor, said factor has a protagonist effect on the response and in the opposite case there is an antagonist effect. To confirm this, the analysis of variance made by the Statgraphics software is presented in electronic supplementary material, tables S3 and S4 for CRP and CRB, respectively.

**Figure 2.**
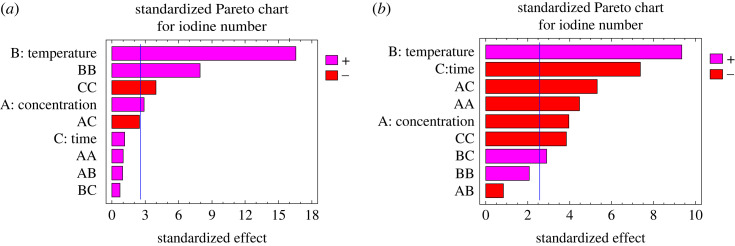
(*a*) Pareto diagram of CRP and (*b*) Pareto diagram of CRB.

**Figure 3.**
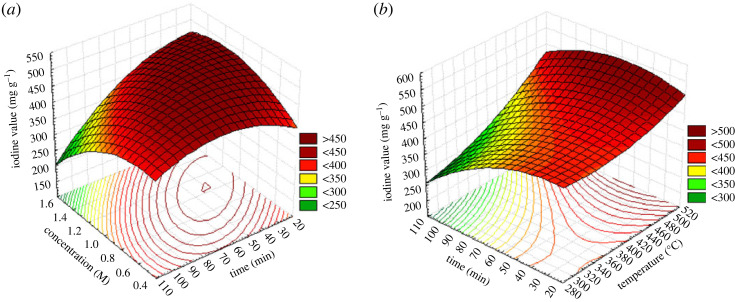
Response surface of CRB (*a*) concentration and time with fixed temperature 400°C and (*b*) iodine number according to the temperature and time with a fixed concentration of 1 mol l^−1^.

**Figure 4.**
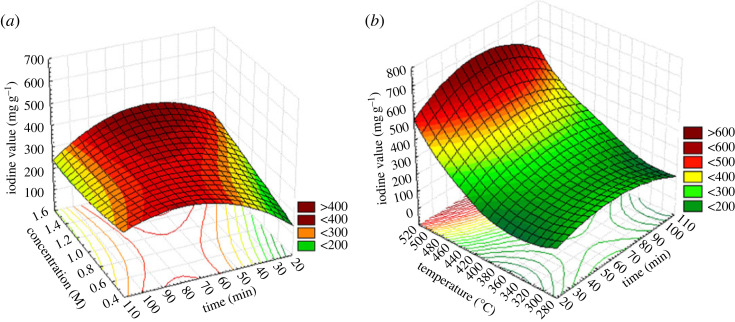
Response surface of CRP (*a*) concentration and time with fixed temperature 400°C and (*b*) iodine number according to the temperature and time with a fixed concentration of 1 mol l^−1^.

The values of the correlation coefficients *R*^2^ and *R*^2^ adjusted are close to unity meaning that the values observed experimentally are close to those predicted by the model, which makes it possible to validate the model. The expressiveness of the coefficients is verified by the Student test which specifies that a coefficient is said to be considerable if it is greatly different from zero for a degree of confidence of 95%. In other words, a coefficient is significant if its observed *p*-value is less than 0.05 [27]. Otherwise the coefficient has little or no influence on the response. In the case of the optimization of the preparation of CRP, two of the three factors investigated have a remarkable and positive influence on the iodine number; this is firstly the carbonization temperature and then the concentration of the activating agent; moreover the influence of the three factors on the optimization of CRBs is significant, However, only the carbonization temperature has a positive effect on the iodine number. These results are confirmed by the Pareto diagram (figure 2*a*,*b*) whose significant effect is observed when it is greater than 2.6 normalized units on the *x*-axis of the diagram. Figures 3 and 4 show the evolution of the iodine number according to the most significant interaction (concentration and time with a fixed temperature of 400°C and temperature and time with fixed concentration of 1 mol l^−1^) for CRB and CRP, respectively. For the two carbons used for iodine adsorption, the carbonization temperature had a positive effect on the response. This may be due to the fact that, at high temperatures, dehydration occurs in the materials leading to the loss of water in the gaseous form. This favours the formation of ACs of large specific surface areas and a marked increase in the porosity. These results are in agreement with those obtained by Amola *et al.* [15].

According to figures 2–4, it is also found that the concentration of the activating agents has a significant effect on the microporosity of said carbons; however, this factor is positive for the adsorption of iodine on CRP and negative on the CRB. This can be explained by the fact that, in either case, the increase in the concentration of the activating agent promotes the creation of micropores up to a certain concentration threshold. Past this threshold, a higher concentration could lead to an enlargement of these pores, thus leading to meso and macropores. This negative effect can also come from a possibility of clogging the pores by the activating agent which has not undergone a reaction. However, the combined action of high concentration and high temperature increases the pores of AC, thereby leading to high iodine number values. Similar results were found by Lekene *et al.* [28].

We notice that the ACs modified with H_3_PO_4_ presented a better microporosity compared to those modified with NaOH, which is explained by the fact that H_3_PO_4_ is a dehydrating agent which prevents the formation of tar as well as any liquids that can block the pores of ACs [29].

The ideal conditions for the preparation of the ACs were obtained from the maximum iodine number. Table 3 shows the combination of factor levels which maximizes iodine number over the indicated region for CRP and CRB, respectively. We note from this table that the values of the optimal iodine numbers predicted by the Statgraphics plus 5.0 software are substantially equal to those found experimentally.

**Table 3.** Validation of the experimental model.

activated carbons	preparation conditions	IN (mg g^−1^)		
		experimental value	predicted value	residue
CRP	A (mol l^−1^): 1.5	641.29	623.706	17.584
	B (°C): 500			
	C (min): 62			
CRB	A (mol l^−1^): 0.85	575.15	547.183	27.85
	B (°C): 500			
	C (min): 59			

### 3.2. Physical analysis

#### 3.2.1. Energy dispersive X-ray analysis

**Figure 5.**
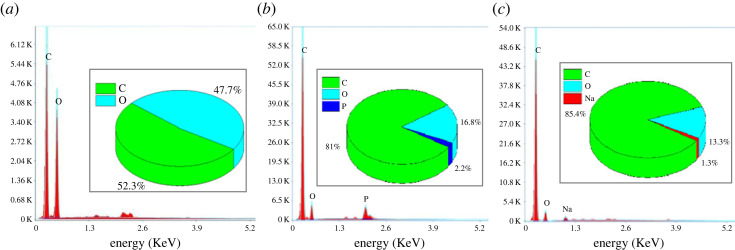
(*a*) EDX analysis of CRN, (*b*) EDX analysis of CRP and (*c*) EDX analysis of CRB.

The results of the EDX analyses of the raw materials and the ACs provided us with information on the elemental composition of the respective materials, in order to confirm that the chosen materials are good precursors for the preparation of AC. The raw material was analysed by EDX in order to determine its chemical composition and demonstrate its high carbon content. The AC was also analysed by EDX in order to see their elemental composition and compare with those of the raw materials. This will allow us to see the effects of activating agent and carbonization on the raw material. Figure 5 shows the spectra obtained from the EDX analysis of the raw material (CRN), CRP and CRB, respectively.

According to these results, it can be seen that the CRN is essentially made up of carbon and oxygen. Moreover the high percentage of carbon in CRN proves that it is a good precursor for the preparation of AC. A decrease in oxygen content can also be observed which may be due to high temperature dehydration. In the elemental composition of CRP and CRB, the elements phosphorus and sodium appear, respectively. The presence of these elements may result from the reaction of phosphate and sodium ions from the activating agents with the precursor. It could, therefore, be said that chemical activation with H_3_PO_4_ and NaOH as activating agents for CRP and CRB, respectively, has indeed been carried out.

#### 3.2.2. Scanning electron microscopy analysis

**Figure 6.**
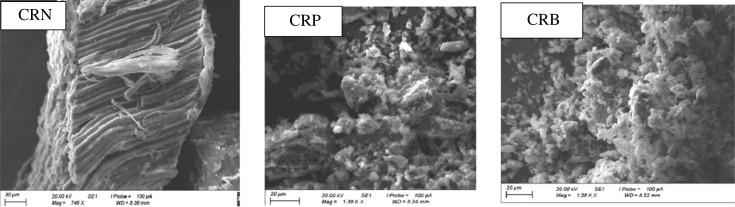
SEM images of the CRN, CRP and CRB.

Figure 6 shows the photographs obtained by scanning electron microscopy (SEM) of the CRN, CRP and CRB.

We can notice from these images significant influence of activation and carbonization on the development of the porous structure. The microphotography of the surfaces of the ACs shows a disorganized structure with a distribution of pores of diverse sizes and forms, hence the heterogeneity of the surfaces of the different ACs. Images of CRP and CRB obtained by SEM show a scabrous semblance of the surfaces, the presence of a multitude of fine particles attached to the ACs. This could correspond to the impurities acquired during the preparation of the ACs such as the ashes but also lasting of the vegetable source of the ACs. From the photographs, it can be seen that the CRP developed more micropores than the CRB. Knowing that the more porous a material, the greater its specific surface area and therefore more sites for the attachment of pollutants [30]. We can, therefore, say that H_3_PO_4_ as an activating agent develops more micropores than NaOH (this has moreover been confirmed by the results of iodine number). This conclusion was endorsed by Balogoun *et al.* [31].

#### 3.2.3. XRD of ACs

**Figure 7.**
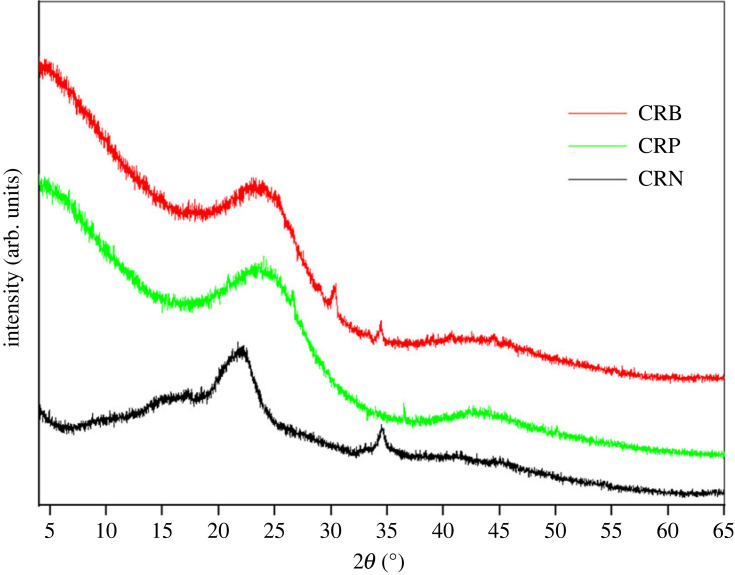
Diffraction patterns of: CRN, CRP and CRB.

Figure 7 presents the results of the XRD analysis of the CRN, CRP and CRB, respectively.

The difference between CRN and different ACs proves that chemical activation and carbonization have clearly modified the structure of the raw material. The diffraction patterns of the two ACs have the same shape and show similar peaks at 2*θ* = 22° and 2*θ* = 42° which are attributed, respectively, to the presence of carbon and dehydrated hemicellulose [32,33]. We can also note the presence of the background noise signals corresponding to the ACs powder, thus revealing an absence of crystallinity and therefore an amorphous structure of the ACs which is an advantageous property for well-defined porous adsorbent [34]. This can be explained by the fact that subjection of CRN to high temperatures contributed to the breaking of the chemical bonds [35]. The structure of CRN is less amorphous compared to the two ACs due to the carbonization. This proves that activation and carbonization of CRN was efficient.

#### 3.2.4. Raman spectroscopy of ACs

**Figure 8.**
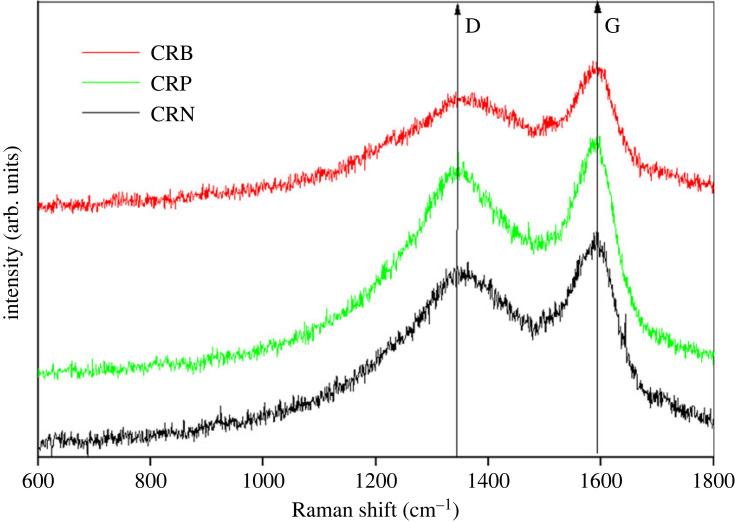
Raman spectra of CRP and CRB.

Analysis of the Raman spectrum obtained for CRP and CRB show wide and intense absorption bands around 1350 cm^−1^ and around 1586 cm^−1^ (figure 8).

The band present around 1350 cm^−1^ is called D band and is characteristic of the disorder within the structure of ACs (amorphous carbon). The band present around 1586 cm^−1^ is the G band which is attributed to the doubly degenerate vibration mode of E_2_g symmetry coming from the graphite layers (sp^2^ carbon) [36]. The ratios of the intensity of the D (*I*_D_) band to that of the G band (*I*_G_) of both CRP and CRB have been determined. These ratios being 0.90 and 0.96 for CRB and CRP, respectively, indicate that CRP have a more amorphous and therefore less ordered structure than CRB; knowing that, the more the *I*_D_/*I*_G_ ratio decreases, the more the structure of the material is ordered [37]. These observations are in agreement with the results obtained with SEM and XRD analyses.

#### 3.2.5. Specific surface area

**Figure 9.**
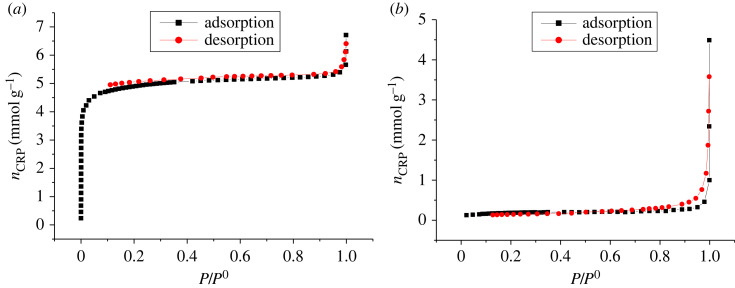
(*a*) Adsorption–desorption curve of N2 at 77 K on CRP and (*b*) adsorption–desorption curve of N2 at 77 K on CRB.

**Figure 10.**
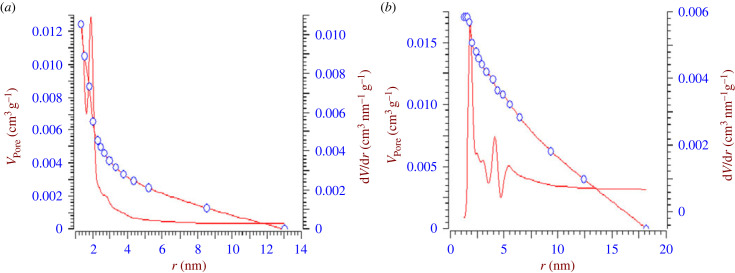
Size distribution of CRP (*a*) and CRB (*b*).

The adsorption–desorption isotherms of N_2_ at 77 K on the ACs were obtained by plotting *n*_adsorb_ = *f*(*P*_0_/*P*) and *n*_desorb_ = *f*(*P*_0_/*P*). The data that allowed the realization of these curves are contained in the electronic supplementary material tables S5 and S6. These isotherms are shown in figures 9 and 10 for CRB and CRP, respectively.

According to the classification of Brunauër *et al.* in 1940 and the IUPAC in 2015, the CRB presents a type III isotherm which confirms, as in the previous results, the macroporosity of the ACs modified with NaOH (CRB). This type of isotherm resulting from the adsorption of N_2_ by the CRB Confers has a lower adsorption at low pressure which could be explained by the weak attraction between the CRB and the N_2_ and a high interaction between the molecules of N_2_. The absence of the micropores noted in the CRB could be due to the strong alkalinity of its activating agent which is NaOH. Indeed the latter would have reacted violently with the CRN with the consequence of an enlargement of the pores [38]. Moreover, the isotherms obtained for the adsorption of N' on CRP indicate a strong adsorption at low temperature then a relatively gradual increase in the quantity adsorbed. These observations are characteristics of type I which indicate monolayer adsorption on a microporous material isotherm and a hysteresis which is due to condensation of the pores. We can also emit coexistence between the micropores and macropores. The specific surface area of ACs calculated by the BET method, the total pore volume, the micropore volume calculated from Durbinin–Radushkevich theory and the pore diameter estimated from the ratio of the total pore volume to the specific surface area are presented in table 4.

**Table 4.** Summary report of micrometrics analysis for CRP and CRB.

ACs	*S*_BET_ (m^2^ g^−1^)	total volume (cm^3^ g^−1^)	micropores			mesopores		
			*S*_micro_ (m^2^ g^−1^)	*V*_micro_ (cm^3^ g^−1^)	Dp (nm)	*S*_meso_ (m^2^ g^−1^)	*V*_meso_ (cm^3^ g^−1^)	Dp (nm)
CRP	501.23	115.16	509.05	0.1807	1.906	10.72	0.0125	1.9066
CRB	15.055	3.4589	27.532	0.0098	0.690	7.2303	0.017	1.9155

The specific surface areas obtained by BET confirm that the CRP is more microporous than CRB. These results are in agreement with results of iodine number found above. The pore size distribution was obtained from the desorption branch by the Barett–Joyner–Halenda (BJH) method. The results of CRP and CRB are grouped in figure 10.

#### 3.2.6. FTIR spectra

**Figure 11.**
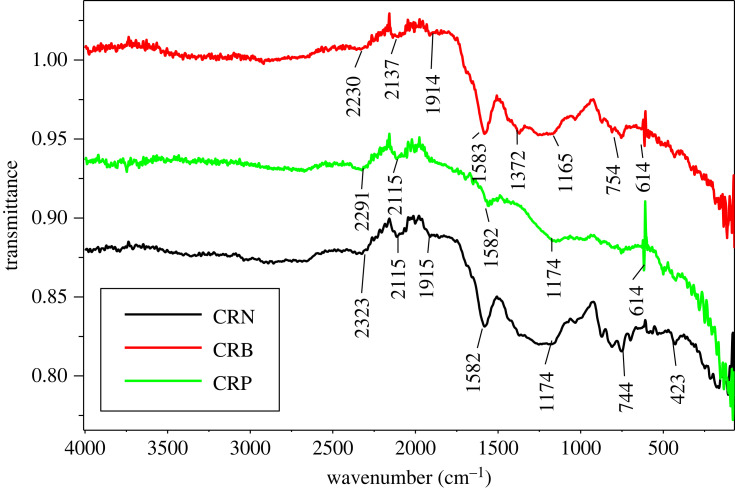
FTIR spectra for biomass (CRN) and activated carbons (CRB and CRP).

Figure 11 shows the FTIR spectra recorded in the 4000–100 cm^−1^ region.

The spectra of the different materials obtained by FTIR spectroscopy show a weak peak around 2923 cm^−1^ for CRN which is characteristic of the valence vibration of the C–H bond [39]. This peak is not pronounced on the spectra of the prepared ACs. This can be explained by the fact that chemical activation and carbonization have contributed to the breaking of these bonds and thus promote the formation of oxygenated functional groups [15], the peaks observed at 1914 cm^−1^ are attributed to the C–O bond stretching vibrations of ketones. Between 2137 and 2230 cm^−1^ the vibration bands observed are attributed to the presence of O–C–O groups or phosphanate groups. Around 1582 cm^−1^, the elongation vibration bands are assigned to the C–O bond of the ester and lactone functions. The peak observed at 1372 cm^−1^ could correspond to the deformation in the symmetrical plane of the C–H bond of the methyl groups. The peaks between 1165 and 1200 cm^−1^ are attributed to stretching vibrations of the C–O bond, carboxyl functions and/or ethers. Between 800 and 650 cm^−1^ we have the C–H bond of aromatic polynuclear systems.

#### 3.2.7. Characterization of the activated carbons: pH, pH at zero point charge, bulk density, moisture content, activated carbon yield and Boehm titration

The physico-chemical properties obtained for the various ACs are presented in table 5.

**Table 5.** Some physico-chemical characteristics of CRP and CRB.

	pH	pH_pzc_	moisture content (%)	bulk density (g ml^−1^)	carbon yield (%)
CRP	3.1	2.11	4	844	46.36
CRB	9.814	9.71	5	576	36.34

The pH of the ACs gives information about their basicity or the acidity. We can, therefore, see from the results obtained that the CRP is acidic (pH = 3.1) while the CRB is basic (pH = 9.814). These could be due to the acidic and basic character of the activating agents used for the preparation of CRP and CRB, respectively.

**Figure 12.**
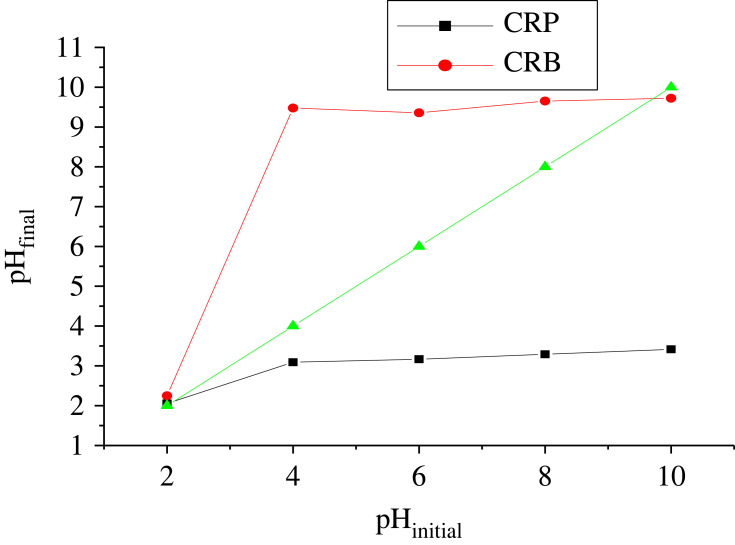
Determination of the pH at the point of zero charge (pHpzc) for CRP and CRB.

The pH for which the ACs are electrically neutral are 2.11 and 9.71 for CRP and CRB, respectively. Thus, if we have a solution whose pH is higher than the pH_pzc_, the electrostatic attraction is favourable for the pollutant in cationic form because the surface of the material is negatively charged. On the other hand, if we have a solution whose pH is lower than pH_pzc_, the electrostatic attraction is propitious for an anionic pollutant because the surface of the material is positively charged. Figure 12 shows the plot of pH_final_ = *f*(pH_initial_) [16,40]. The data that allowed the realization of these curves are contained in electronic supplementary material, table S7 [41].

According to the American Society for Test and Material (ASTM D2867), an AC is best if it has a moisture content of 5% or less. The moisture content of CRB and CRP being 5% and 4% confirm the good quality of these two ACs produced. These assessments are confirmed by the results obtained from the bulk density. The bulk densities being 834 g ml^−1^ and 576 g ml^−1^ for CRP and CRB, respectively, show that both ACs are above 475 g ml^−1^ which is the bulk density limit set by ASTM for ACs [12].

The carbon yields of CRP (46.36%) and CRB (36.34%) attest to the good quality of precursor for the preparation of ACs because the loss of volatile matter is limited [42,43]. Since CRP has a higher yield than CRB, it can be said that H_3_PO_4_ reduces the loss of volatile matter more than NaOH. The same remarks were observed by Zhao *et al*. in 2011 [44].

The quantification of the surface functions of the different ACs obtained by Boehm analysis are recorded in table 6.

**Table 6.** Quantification of surface functions by Boehm titration.

activated carbons	carboxylic	lactone	phenol	total acid	total base
CRP	0.06	0.18	0.84	1.08	0.12
CRB	0.00	0.14	0.1	0.24	0.96

These results prove the acid character of CRP and the basic character of CRB. These are in agreement with the pH values obtained.

## 4. Conclusion

From this study, which focused on the optimization of the preparation of highly porous ACs from puck shells by the RSM, it appears that puck shells are good precursors for the preparation of AC. The ACs were prepared by chemical activation, using H_3_PO_4_ and NaOH as the activating agents. The Box–Behnken model was used to study the influence of temperature of carbonization, concentration of activating agent and carbonization time. The considered response was iodine number which was 641.29 mg g^−1^ for CRP and 575.15 mg g^−1^ for CRB. The optimum conditions generated from the Statgraphics 5 plus software were 1.5 M; 500°C and 62 min for CRP and 0.85 M; 500°C, 58.28 min for CRB. The results of pH Boehm titration and of FTIR indicate that the AC CRP is acidic while CRB is basic. The analysis of physisorption of N_2_ made it possible to quantify the microporous surface of the different ACs which was 509.05 m^2^ g^−1^ and 27.532 m^2^ g^−1^ for CRP and CRB, respectively. These results show that CRP developed better surface properties compared to CRB.

## Data Availability

The data presented in this study, together with relevant code for this research work, are stored in Mendeley repository [41] (https://doi.10.17632/dm92gjxx8y.1). The data are provided in the electronic supplementary material and this part of data is allowed to be copied, distributed, transmitted and adapted by anyone [41] Supplementary material is available online [45].

## References

[1] Xiaomi S, Mingming W, Songwen Y, Feiyun S, Jiaju D, Yiping R, Bin Z, Desong H. 2020 Tetrabromobisphenol A adsorption by active carbon: influential factors and kinetics. E3S Web Conf. **144**, 01013. (10.1051/e3sconf/202014401013)

[2] Wang J, Wu F, Wang M, Qiu N, Liang Y, Fang S, Jiang X. 2010 Preparation of activated carbon from a renewable agricultural residue of pruning mulberry shoot. Afr. J. Biotechnol. **19**, 2762-2767.

[3] Azharul I, Ahmed M, Khanday W, Asif M, Hameed B. 2017 Mesoporous activated carbon prepared from NaOH activation of rattan (*Lacosperma secundiflorum*) hydrochar for methylene blue removal. Ecotoxicol. Environ. Saf. **138**, 279-285. (10.1016/j.ecoenv.2017.01.010) 28081490

[4] Basta A, Fierro V, Saied H, Celzard A. 2011 Effect of deashing rice straws on their derived activated carbons produced by phosphoric acid activation. Biomass Bioenergy **35**, 1954-1959. (10.1016/j.biombioe.201101.043)

[5] Mohammad J, Chia C, Suffian M, Sarani Z, Mohd S, Mahammad H. 2020 Rice husk activated carbon with NaOH activation: physical and chemical properties. Sains Malays. **49**, 2261-2267. (10.17576/jsm--2020-4909-23)

[6] Tchuifon DR, Anagho SG, Njanja E, Ghogomu JN, Ndifor-Angwafor NG, Kamgaing T. 2014 Equilibrum and kinetic modeling of methyl orange adsorption from aqueous solution using rice husk and egussi peeling. Int. J. Chem. Sci. **12**, 741-761.

[7] Ndifor-Angwafor GN, Bopda A, Tchuifon DR, Ngakou SC, Kuete TH, Anagho SG. 2017 Removal of paracetamol from aqueous solution by adsorption onto activated carbon prepared from rice husk. J. Chem. Pharm. Res. **9**, 56-68.

[8] Khanday W, Marrakchi F, Asif M, Hameed B. 2017 Mesoporous zeolite–activated carbon composite from oil palm ash as an effective adsorbent for methylene blue. J. Taiwan Inst. Chem. Eng. **70**, 32-41. (10.1016/j.jtice.2016.10.029)

[9] Azharul I, Sabar S, Benhouria A, Khanday W, Asif M, Hameed B. 2017 Nanoporous activated carbon prepared from karanj (*Pongamia pinnata*) fruit hulls for methylene blue adsorption. J. Taiwan Inst. Chem. Eng. **74**, 96-104. (10.1016/jtice.2017.01.016)

[10] Ndi NJ, Ketcha MJ, Anagho GS, Ghogomu NJ, Belibi PD. 2014 Physical and chemical characteristics of activated carbon prepared by pyrolysis of chemically treated Cola nut (*Cola acuminata*) shells wastes and its ability to adsorb organics. Int. J. Adv. Chem. Technol. **3**, 1-13.

[11] Kuete TI-H, Tchuifon TR, Doungmo G, Anagho SG. 2018 Preparation and characterization of activated carbons from bitter kola (*Garcinia kola*) nut shells by chemical activation method using H_3_PO_4_; KOH and ZnCl_2_. Chem. Sci. Int. J **23**, 1-15. (10.9734/CSJI/2018/43411)

[12] Ngakou CS, Ngomo HM, Anagho GS. 2018 Batch equilibrium and effects of ionic strength on kinetic study of adsorption of phenacetin from aqueous solution using activated carbon derived from a mixture of ayous sawdust and Cucurbitaceae peelings. Curr. J. Appl. Sci. Technol. **26**, 1-24. (10.9734/CJAST/2018/37300)

[13] Amola LA, Kamgaing T, Tchuifon DR, Atemkeng CD, Anagho SG. 2020 Activated carbons based on shea nut shells (*Vitellaria paradoxa*): optimization of preparation by chemical means using response surface methodology and physicochemical characterization. J. Mater. Sci. Chem. Eng. **8**, 53-72. (10.4236/msce.2020.88006)

[14] Kamgaing T, Doungmo G, Tchieno M, Kouonang J, Ketcha J. 2017 Kinetic and isotherm studies of bisphenol A adsorption onto orange albedo (*Citrus sinensis*): sorption mechanism based on the main albedo components vitamin C, flavones glycosides and caortenoids. J. Environ. Sci. Health A **52**, 757-769. (10.1080/10934529.2017.1303315) 28394738

[15] Amola LA, Kamgaing T, Tagne RFT, Atemkeng CD, Kuete I-H, Anagho SG. 2022 Optimized removal of hydroquinone and resorcinol by activated carbon based on shea residue (*Vitellaria paradoxa*): thermodynamics, adsorption mechanism, nonlinear kinetics, and isotherms. J. Chem. **2022**, 15. (10.1155/2022/1125877)

[16] Atemkeng DC, Kamgaing T, Tchuifon TDR, Doungmo G, Amola AL, Kamdem TA, Anagho SG. 2020 Chemical preparation and physicochemical characterization of powdered activated carbons based on Safou (*Dacryodes edulis*) seeds. J. Mater. Environ. Sci. **11**, 896-910.

[17] Şahin O, Cafer S, Ayhan AC, Orhan B. 2016 The pyrolysis process of biomass by two-stage chemical activation with different methodology and iodine adsorption. Energy Sources A: Recovery Util. Environ. Eff. **38**, 1756-1762. (10.1080/15567036.2014.956195)

[18] Bopda A, Tchuifon DR, Nche GN, Doungmo G, Anagho SG. 2019 Non-linear equilibrium and kinetic study of the adsorption of 2,4-dinitrophenol from aqueous solution using activated carbon derived from a olives stones and cotton cake. Afr. J. Environ. Sci. Technol. **3**, 365-380. (10.5897/ajest2019.2717)

[19] Romero-Anaya AJ, Ouzzine M, Ródenas MA, Linares-Solano A. 2014 Spherical carbons: synthesis, characterization and activation processes. J. Carbon **68**, 296-307. (10.1016/j.carbon.2013.11.006)

[20] Bopda A et al. 2022 Ferromagnetic biochar prepared from hydrothermally modified calcined mango seeds for fenton-like degradation of indigo carmine. Carbon **8**, 81. (10.3390/c8040081)

[21] Atemkeng CD, Tamo AK, Doungmo G, Amola LA, Ngouoko J, Kamgaing T. 2022 Thermodynamic, nonlinear kinetic, and isotherm studies of bisphenol A uptake onto chemically activated carbons derived from Safou (*Dacryodes edulis*) Seeds. J. Chem. **2022**, 17. (10.1155/2022/7717148)

[22] Goupy J. 1996 Méthode des plans d'expériences: optimisation du choix des essais et de l'interprétation des résultats. Paris, France: Edition Dunod.

[23] Azizi A, Mohammad R, Moghaddam A, Arami M. 2011 Application of response surface methodology for optimization of reactive blue 19 dye removal from aqueous solutions using pulp and paper sludge. Fresenius Environ. Bull. **20**, 929-938.

[24] ASTM ‘Method D4607-94(99). 1994 Standard test method for determination of iodine number of activated carbon. Washington, DC: ASTM International.

[25] Danish M, Ahmad T. 2018 A review on utilization of wood biomass as a sustainable precursor for activated carbon. production and application. Renew. Susaint. Energy Rev. **87**, 1-21. (10.1016/j.rser.2018.02.003)

[26] Boehm HP. 2002 Surface oxides on carbon and their analysis: a critical assessment. Carbon **40**, 145-149. (10.1016/S0008-623(01)00165-8)

[27] Student WSG. 1908 The probable error of a mean. Biometrika **6**, 1-25. (10.230/2331554)

[28] Lékéné RBN, Nsami JN, Rauf A, Kouotou D, Belibi PDB, Bhanger MI, Mbadcam JK. 2018 Optimization conditions of the preparation of activated carbon based Egusi (*Cucumeropsis mannii* Naudin) seed shells for nitrate ions removal from wastewater. Am. J. Anal. Chem. **9**, 439-463. (10.4236/ajac.2018.910034)

[29] Lua AC, Yang T. 2005 Characteristics of activated carbon prepared from pistachio-nut shell by zinc chloride activation under nitrogen and vacuum conditions. J. Colloid Interface Sci. **290**, 505-513. (10.1016/j.jcis.2005.04.063) 16002081

[30] Hazourli S, Ziata M, Hazourli A, Cherifi M. 2009 Characterization of activated carbon prepared from lignocellulosic natural residue: example of date stones. Phys. Procedia **2**, 1039-1043. (10.1016/j.phpro.2009.11.060)

[31] Balogoun CK, Bawa ML, Osseni S, Aina M. 2015 Préparation des charbons actifs par voie chimique à l'acide phosphorique à base de coque de noix de coco. Int. J. Biol. Chem. Sci. **9**, 563-580. (10.4314/ijbcs.v9i1.48)

[32] Xinyang L, Wu G. 2021 Porous carbon from corn flour prepared by H3PO4 carbonization with KOH activation supercapacitors. J. Power Eng. **9**, 18-25. (10.4236/jpee.2021.98002)

[33] Reffas A, Bernadet V, David B, Reinert L, Bencheich M, Dubois M, Batisse N, Duchaux L. 2009 Carbons prepared from coffee ground by H3PO4 activation: characterization and adsorption of methylene blue and Nylosan red N-2RBL. J. Hazard. Mater. **175**, 779-788. (10.1016/j.jhazmat.2009.10.076) 19942347

[34] Kuete TI-H, Raoul Donald T, Christian SN, Ndifor-Angwafo N, Anagho G. 2022 Adsorption of indigo carmine onto chemically activated carbon derived from the Cameroonian agricultural waste Garcinia cola nut shells and desorption studies. J. Chem. **2022**, 19. (10.1155/2022/1236621)

[35] Huang W, Li D, Liu Z-Q, Tao Q. 2014 Kinetics, isotherm, thermodynamic and adsorption mechanism studies of La(OH)3 modified exfoliated vermiculites as highly efficient phosphate adsorbents. Chem. Eng. J. **236**, 191-201. (10.1016/j.cej.2013.09.077)

[36] Kumar P, Divya N, Kumar J. 2021 Study on the physico-chemical properties of reduced graphene oxide with different degrees of reduction temperature. J. Iran. Chem. **18**, 201-211. (10.1007/s13738-020-02014-w)

[37] Maslova OA. 2014 Spectroscopie et imagerie Raman de matériaux inhomogènes. Doctoral Thesis, University of Orléans, France.

[38] Brunauer S, Deming LS, Deming WE, Teller E. 1940 On a theory of the van der Waals adsorption of gases. J. Chem. Soc. **62**, 1723-1732. (10.1021/ja01864a025)

[39] Nko'o AM, Avom J, Mpon R. 2016 Évaluation des propriétés de charbons actifs de résidus de Moabi (*Baillonella toxisperma* Pierre) par adsorption d'iode en solution aqueuse. J. Water Sci. **29**, 51-60. (10.7202/1035716ar)

[40] Pongener C, Kibami D, Rao K, Goswamee RL, Sinha D. 2015 Synthesis and characterization of activated carbon from the biowaste of the plant *Manihot esculenta*. Chem. Sci. Trans. **4**, 59-68. (10.7598/cst2015.958)

[41] Tagne R. 2023 Optimized preparation of activated carbon with high porosities based on puck shells (*Afrostyrax lepidophyllus*) by response surface methodology and physico-chemical characterization. Mendeley Data V1 **2**, 526–534. (10.17632/dm92gjxx8y.1) PMC10698491 38077221

[42] Abdulsalam M, Hasfalina CM, Mohamed HA, Abd Karim SF, Faiez MS. 2018 Microwave irradiated coconut shell-activated carbon for decolourisation of palm oil mill effluent (POME). Food Res. **2**, 526-534. (10.26656/fr.2017.2(6).103)

[43] Ekta R, Monita AB, Archana RC. 2023 Study of synthesis and characterization of raw bagasse, its char and activated carbon prepared using chemical additive. Water Sci. Technol. **87**, 2233–2249. (10.2166/wst.2023.134) 37186627

[44] Zhao W, Fierro V, Zlotea C, Aylon E, Izquierdo M, Latroche M, Celzard A. 2011 Optimization of activated carbons for hydrogen storage. Int. J. Hydrog. Energy **36**, 11 746-11 751. (10.1016/j.ijhydene.2011.05.181)

[45] Nzetchuen Kouahou G, Fotsop CG, Adoum Amola L, Donlifack Atemkeng C, Kamdem Tamo A, Teikam Kenda G, Tiegam Tagne RF, Kamgaing T. 2023 Optimized preparation of activated carbon with high porosities based on puck shells (*Afrostyrax lepidophyllus*) by response surface methodology and physico-chemical characterization. Figshare. (10.6084/m9.figshare.c.6956874) PMC10698491 38077221

